# Supplementary material for: Clustering of 27,525,663 Death Records from the United States Based on Health Conditions Associated with Death: An Example of Big Health Data Exploration
Source: J Clin Med. 2019 Jun 27;8(7):922. doi: 10.3390/jcm8070922 (PMC6678953; doi:10.3390/jcm8070922)
Supplement: Supplementary file 1 [file jcm-08-00922-s001.zip › jcm-532622(4).docx]

Supplementary A

*Data Collection*

The data for this study was downloaded in csv-Format from http://www.nber.org/data/vital-statistics-mortality-data-multiple-cause-of-death.html on November 14th, 2017. It originates from the Multiple Cause-of-Death Mortality Data of the National Vital Statistics System of the National Center for Health Statistics in the United States of America. In this data set every death occurring in the United States (since 1959) and U.S. Territories (since 1994) is listed along information abstracted from death certificates filed in vital statistics offices. This study uses all data records for the United States (U.S. Territories excluded) for the years 2006 to 2016. This restriction was made to ensure good comparability of all analyzed data records by excluding effects of changed coding schemes, changed attributes and coding trends. The attributes accessible in the data set are listed in Table A1.

*Definitions*

Health conditions (the variables *enicon_1* to *enicon_30* from the original data set) and underlying cause of death (*ucod*) where given in the ICD-10 coding scheme (International Classification of Diseases, 1992 Revision, Volume 1 for years 2006–2009 and International Classification of Diseases, 2004 Revision, Volume 1 for years 2010–2014) in the data source. These codes were aggregated into 346 disease categories (Table A2).The aggregations were made from a data analytic point of view with additional consideration of medical connections and the structure of the ICD10 codes. First all codes in the entity variables of the data set where listed and their frequency of occurrence was considered. Relatively frequent codes (>0.05% prevalence in the data set) were considered important for the analysis. Less frequent codes where added to similar more frequent ones, whenever possible. When this was not possible, a more general group was considered for specific codes or codes where aggregated into leftover categories, such as “Other infection” or “Other metabolic disease”.

The information about the education level of the deceased persons was given in two different coding schemes (*educ1989* and *educ2003*) for different years. The generalization made to combine the information of the different schemes is provided in Table A3.

The coded variable age was recoded to give age in completed years. The variable *racer5* of the original data set was used as race variable. For resident status, the two categories 2 (*intrastate residents*) and 3 (*interstate residents*) were combined into non-local US resident, while other people were categorized as local residents (resident of the state and county in which he or she died) or foreign residents. Place of death was given as: hospital (inpatient); hospital (outpatient); hospital (death on arrival); home; hospice facility; nursing home; and other. Finally, a multimorbidity indicator was defined, which is 1 if the number of different health conditions (*eanum*) is two or more (*ucod* and one or more additional entity codes), and 0 otherwise.

Supplementary B

The ‘Gastrointestinal Cancer’ Cluster (n = 1,586,848)

The top-5 most prevalent health conditions associated with death in this cluster were: (1) colon cancer (27.6%); (2) pancreas cancer (24.1%); (3) liver cancer (20.0%); 4) ovarian cancer (10.3%); and (5) cardiac arrest (9.1%). Moreover, the top-5 most prevalent underlying primary causes of death were: (1) colon cancer (24.8%); (2) pancreas cancer (23.5%); (3) liver cancer (12.7%); (4) ovarian cancer (9.6%); and (5) stomach cancer (7.4%). Compared to the whole population, the deceased in this cluster: had a significantly lower mean age at the time of death; had a higher proportion of women; were more often black or Asian/Pacific islander; were more likely to have received tertiary education; were more often married or divorced; were more often local residents; more often died at home; and were more likely to have died of a natural cause. Moreover, the deceased had less health conditions associated with death; and a lower prevalence of multimorbidity.

The ‘Stroke’ Cluster (n = 1,507,867)

The top-5 most prevalent health conditions associated with death in this cluster were: (1) unspecified stroke (51.1%); (2) cerebral hemorrhage (26.9%); (3) hypertension (26.3%); (4) other cerebrovascular disease (17.3%); and (5) cardiac arrhythmia (12.1%). Moreover, the top-5 most prevalent underlying primary causes of death were: (1) unspecified stroke (32.9%); (2) cerebral hemorrhage (21.7%); (3) other cerebrovascular disease (8.6%); (4) atherosclerotic heart disease (6.7%); and (5) cerebral infarction (5.1%). Compared to the whole population, the deceased in this cluster: had a significantly higher mean age at the time of death; had a higher proportion of women; were more likely to be black or Asian/Pacific islander; more often received only primary education; were more often widowed; more likely to be a non-local US resident or foreign resident; more often died in hospital as inpatient, hospice facility or nursing home; and more often died of natural causes. Moreover, the deceased had less health conditions associated with death; and a lower prevalence of multimorbidity.

The ‘Atherosclerotic Heart Disease’ Cluster (n = 1,389,311)

The top-5 most prevalent health conditions associated with death in this cluster were: (1) atherosclerotic heart disease (82.2%); (2) hypertension (29.9%); (3) cardiac arrest (27.0%); (4) lipidaemia (22.5%); and (5) diabetes mellitus (20.5%). Moreover, the top-5 most prevalent underlying primary causes of death were: (1) atherosclerotic heart disease (70.0%); (2) diabetes mellitus (8.6%); (3) lipidaemia (4.9%); (4) myocardial infarction (3.6%); and (5) other acute ischemic heart disease (3.1%). Compared to the whole population, the deceased in this cluster: had a significantly higher mean age at the time of death; a higher proportion of men; were more likely to be black or Asian/Pacific islander; more often received secondary but no tertiary education; were more likely to be widowed or divorced; more likely to be a local resident or foreign resident; more often died in the hospital (as outpatient, in emergency room or dead on arrival), at home or in a nursing home; and more often died of natural causes. Moreover, the deceased had less health conditions associated with death; and a lower prevalence of multimorbidity.

The ‘Neurodegenerative Disease’ Cluster (n = 1,309,617)

The top-5 most prevalent health conditions associated with death in this cluster were: (1) Alzheimer’s disease (62.7%); (2) Parkinson’s disease (24.4%); (3) hypertension (11.8%); (4) cardiac arrest (10.1%); and (5) atherosclerotic heart disease (9.8%). Moreover, the top-5 most prevalent underlying primary causes of death were: (1) Alzheimer’s disease (53.8%); (2) Parkinson’s disease (16.8%); (3) other degenerative neural disease (6.3%); (4) motor neuron disease (5.5%); and (5) atherosclerotic heart disease (4.1%). Compared to the whole population, the deceased in this cluster: had a significantly higher mean age at the time of death; a higher proportion of women; were more likely to be white; more likely to have received only primary or up to tertiary education; more often widowed; more often local residents; more likely to have died in a nursing home or ‘other’ place (i.e., not in a hospital, hospice or at home); and more often died of natural causes. Moreover, the deceased had less health conditions associated with death; and a lower prevalence of multimorbidity.

The ‘Lung Cancer’ Cluster (n = 1,307,406)

The top-5 most prevalent health conditions associated with death in this cluster were: (1) lung cancer (96.5%); (2) nicotine dependence (41.2%); (3) chronic obstructive pulmonary disease (13.5%); (4) cardiac arrest (7.8%); and (5) respiratory failure (7.6%). Moreover, the top-5 most prevalent underlying primary causes of death were: (1) lung cancer (94.0%); (2) laryngeal cancer (3.0%); (3) chronic obstructive pulmonary disease (1.2%); (4) atherosclerotic heart disease (0.6%); and (5) myocardial infarction (0.2). Compared to the whole population, the deceased in this cluster: had a significantly lower mean age at the time of death; had a higher proportion of men; were more often white; more often received secondary, but no tertiary education; were more likely to be married or divorced; were more often local residents; were more likely to have died at home or in a hospice facility; and more often died of natural causes. Moreover, the deceased had less health conditions associated with death; and a lower prevalence of multimorbidity.

The ‘Other Physical Harm’ Cluster (n = 1,139,837)

The top-5 most prevalent health conditions associated with death in this cluster were: (1) other unspecified injury (56.1%); (2) accidental fall (28.5%); (3) other accident involving motor vehicle (17.3%); (4) femur fracture (15.3%); and (5) accidental exposure to other mechanical force (10.6%). Moreover, the top-5 most prevalent underlying primary causes of death were: (1) accidental fall (23.9%); (2) other accident involving motor vehicle (17.0%); (3) accidental collision of two or more motor vehicles (10.4%); (4) accidental collision of motor vehicle and pedestrian (5.3%); and (5) accidental exposure to other mechanical force (5.0%). Compared to the whole population, the deceased in this cluster: had a significantly lower mean age at the time of death; had a higher proportion of men; were more often white, American Indian or Asian/Pacific islander; more often received tertiary education; more often were never married; were more likely to be non-local US residents or foreign residents; more often died in the hospital (as inpatient, outpatient, in the emergency room or were dead on arrival) or at an ‘other’ place (such as roads or public places); and more often died due to an accident, suicide or homicide. Moreover, the deceased had more health conditions associated with death; and a higher prevalence of multimorbidity.

The ‘Liver Disease’ Cluster (n = 1,131,928)

The top-5 most prevalent health conditions associated with death in this cluster were: (1) liver fibrosis/cirrhosis (31.0%); (2) alcohol dependence (23.4%); (3) gastrointestinal hemorrhage (21.8%); (4) hepatic failure (16.6%); and (5) other liver disease (13.4%). Moreover, the top-5 most prevalent underlying primary causes of death were: (1) alcoholic liver disease (15.5%); (2) liver fibrosis/cirrhosis (14.0%); (3) viral hepatitis (6.7%); (4) gastrointestinal hemorrhage (6.2%); and (5) alcohol dependence (5.8). Compared to the whole population, the deceased in this cluster: had a significantly lower mean age at the time of death; had a higher proportion of men; had a higher proportion of American Indian and Asian/Pacific islander; were more likely to have received secondary but no tertiary education; were more often divorced or never married; had a higher proportion of non-local US residents or foreign residents; more often died in the hospital (inpatient) or in a hospice facility; and more often died of natural causes. Moreover, the deceased had more health conditions associated with death; and a higher prevalence of multimorbidity.

The ‘Myocardial Infarction’ Cluster (n = 907,095)

The top-5 most prevalent health conditions associated with death in this cluster were: (1) myocardial infarction (100%); (2) atherosclerotic heart disease (38.4%); (3) cardiac arrest (16.6%); (4) hypertension (16.0%); and (5) diabetes mellitus (12.8%). Moreover, the top-5 most prevalent underlying primary causes of death were: (1) myocardial infarction (90.3%); (2) diabetes mellitus (5.1%); (3) chronic obstructive pulmonary disease (1.5%); (4) pneumonia (0.7%); and (5) other sepsis (0.5%). Compared to the whole population, the deceased in this cluster: had a significantly higher mean age at the time of death; had a higher proportion of men; had a higher proportion of white; more often had received no tertiary education; were more often married, widowed or divorced; had a higher proportion of local residents and foreign residents; more often died in the hospital (outpatient, emergency room or dead on arrival); and more often died of natural causes. Moreover, the deceased had less health conditions associated with death; and a lower prevalence of multimorbidity.

The ‘Chronic kidney Disease’ Cluster (n = 554,736)

The top-5 most prevalent health conditions associated with death in this cluster were: (1) chronic kidney disease (99.6%); (2) diabetes mellitus (29.3%); 3) heart failure (27.2%); (4) hypertension (26.1%); and (5) atherosclerotic heart disease (26.1%). Moreover, the top-5 most prevalent underlying primary causes of death were: (1) chronic kidney disease (31.4%); (2) diabetes mellitus (17.4%); (3) atherosclerotic heart disease (12.9%); (4) hypertensive renal disease (7.4%); and (5) myocardial infarction (5.0%). Compared to the whole population, the deceased in this cluster: had a significantly higher mean age at the time of death; had a higher proportion of black, American Indian and Asian/Pacific islander; more often received no tertiary education; were more often widowed; had a higher proportion of local residents; more often died in the hospital (inpatient), in a hospice facility or nursing home; and died of natural causes. Moreover, the deceased had more health conditions associated with death; and a higher prevalence of multimorbidity.

The ‘Open wound/suffocation’ cluster (n = 505,920)

The top-5 most prevalent health conditions associated with death in this cluster were: (1) unspecified open wound (51.9%); (2) open head wound (43.4%); (3) intentional self-harm by firearm discharge (42.3%); (4) assault by firearm discharge (25.9%); and 5) asphyxiation (25.3%). Moreover, the top-5 most prevalent underlying primary causes of death were: (1) intentional self-harm by firearm discharge (42.2%); (2) assault by firearm discharge (25.9%); (3) intentional self-suffocation/strangulation (21.1%); (4) assault by sharp object (3.9%); and (5) other accidental threat to breathing (3.5%). Compared to the whole population, the deceased in this cluster: had a significantly lower mean age at the time of death; had a higher proportion of men; black, American Indian and Asian/Pacific islander; more often received secondary or tertiary education; were more often divorced or never married; were more likely to be local residents or foreign residents; more often died in the hospital (outpatient, emergency room or dead on arrival), at home or an ‘other’ place (like public places); and more often died due to suicide or homicide. Moreover, the deceased had more health conditions associated with death; and a higher prevalence of multimorbidity.

The ‘Poisoning’ cluster (n = 497,436)

The top-5 most prevalent health conditions associated with death in this cluster were: (1) intoxication by other medical drug (50.7%); (2) accidental poisoning by other medical drug (37.5%); (3) accidental poisoning by narcotic (35.2%); (4) intoxication by other opioid (24.1%); and (5) other drug dependence (23.1%). Moreover, the top-5 most prevalent underlying primary causes of death were: (1) accidental poisoning by other medical drug (35.4%); (2) accidental poisoning by narcotic (33.1%); (3) intentional self-poisoning by medical drug (11.3%); (4) poisoning by medical drug with undetermined intent (3.8%); and (5) accidental poisoning by alcohol (3.5%). Compared to the whole population, the deceased in this cluster: had a significantly lower mean age at the time of death; had a higher proportion of men; were more likely to be white or American Indian; more often received secondary or tertiary education; were more often divorced or never married; had a higher proportion of local residents and foreign residents; more often died in the hospital (outpatient, emergency room or dead on arrival), at home or an ‘other’ place (like public places); and more often died due to accident or suicide. Moreover, the deceased had more health conditions associated with death; and a higher prevalence of multimorbidity.

**Table S1.** Accessible attributes.

| Topic | Attributes | Explanation |
| --- | --- | --- |
| Age | age,  ageflag,  ager52  ager27  ager12  ager22 | Coded age and recodes. |
| Gender | sex | States gender of the deceased. |
| Education | educ1989, educ2003,  educflag | Educational level of deceased (two different codings). |
| Race/Ethnicity | race,  brace,  raceimp,  racer3,  racer5,  hispanic,  hispanicr | Race and ethnicity in several different classifications. |
| Resident Status | restatus | Describes if deceased was a resident of the state and/or county in which he or she died. |
| Marital Status | marstat | States if deceased was married, divorced, widowed or never married. |
| Date of Death | year,  monthdth,  weekday | Year, Month, Weekday of death. |
| Place of Death | placdth | States at which kind of place (e.g., home, hospital, nursing facility) the death occurred. |
| Manner of Death | mandeath | Natural, accident, suicide or homicide. |
| Autopsy | autopsy | Indicates if an autopsy was performed. |
| Underlying cause of death | ucod,  ucr358,  ucr113,  ucr130,  ucr39 | States what illness or external cause caused all the health conditions, which finally lead to death.  ucod is coded in ICD10 coding scheme (International Classification of Diseases, 1992 Revision, Volume 1 for years 2006 - 2009 and International Classification of Diseases, 2004 Revision, Volume 1 for years 2010 - 2014), ucrXXX are different recodes of ucod. |
| Health conditions | enicon_1, …,  enicon_20,  eanum,  econdp_1, …,  econdp_20,  econds_1, …,  econds_20,  record_1, …,  record_20 | All health conditions mentioned on the death certificate, along with counts of conditions and indicators for position on the death certificate. For entity-axis (as written on the death certificate) and record-axis (processed codes). |
| Method of Disposition | methdisp | Method of disposition of the body (e.g., burial, cremation). |
| Circumstances of injury | injwork,  activity,  injury | Only for injuries. States if injury occurred during work or specific leisure activity and at which kind of place. |

Source: National Center for Health Statistics (2006–2016).

Table S2. Health conditions given in the ICD-10 coding scheme.

Health conditions given in the ICD-10 coding scheme (International Classification of Diseases, 1992 Revision, Volume 1 for years 2006–2009 and International Classification of Diseases, 2004 Revision, Volume 1 for years 2010–2014).

Source: National Center for Health Statistics (2006–2016).

Please see separate table.

**Table S3.** Coding schemes for education level.

|  | Category | educ1989 | educ2003 | Meaning |
| --- | --- | --- | --- | --- |
| Education | Primary | 00–08 | 1 | deceased has only primary education (elementary school, primary school) or no formal education at all |
|  | Secondary | 09–12 | 2–3 | deceased has some secondary education (middle school, junior high school, high school), but not necessary a diploma and no tertiary education |
|  | Tertiary | 13–17 | 4–8 | deceased has some tertiary education (college, university), but not necessarily a degree |

Source: National Center for Health Statistics (2006–2016).

Table S4. All details of the whole sample and the 16 clusters.

Table S4 provides a detailed overview of the socio-demographics (rows 4–35), place of death (rows 37–44), manner of death (rows 46–50), causes of death (rows 52–398), and health conditions at the time of death (rows 404–750) of the whole sample and each cluster. A red-colored cell in the columns that show the *p*-values means that the cluster-specific value is not significantly different from the value of the whole sample.

Source: National Center for Health Statistics (2006–2016).

Please see separate table.


**Figure S1.** The top-25 most prevalent causes of death between 2006 and 2016 in the United States.

**Figure S2.** The top-25 most prevalent health conditions at death between 2006 and 2016 in the United States.

Figure S3. Attribute pictures showing the health conditions at the time of death of the 16 clusters. Red parts of the map consist of high percentages of persons with the health condition as indicated, whereas blue parts consist of low percentages of the health condition as indicated. The exact percentages are given in the bar under the respective map picture.

Please see separate figure.

Figure S4. Attribute pictures showing the causes of death of the 16 clusters. Red parts of the map consist of high percentages of persons with the cause of death as indicated, whereas blue parts consist of low percentages of the cause of death as indicated. The exact percentages are given in the bar under the respective map picture.

Please see separate figure.
