# Supplementary material for: Clustering of 27,525,663 Death Records from the United States Based on Health Conditions Associated with Death: An Example of Big Health Data Exploration
Source: J Clin Med. 2019 Jun 27;8(7):922. doi: 10.3390/jcm8070922 (PMC6678953; doi:10.3390/jcm8070922)
Supplement: Supplementary file 1 [file jcm-08-00922-s001.zip › Figure A4.docx]

**Figure A4 (part 1/20). Self-organizing maps showing the causes of death of the 16 clusters**

**Figure A4 (part 2/20). Self-organizing maps showing the causes of death of the 16 clusters**

**Figure A4 (part 3/20). Self-organizing maps showing the causes of death of the 16 clusters**

**Figure A4 (part 4/20). Self-organizing maps showing the causes of death of the 16 clusters**

**Figure A4 (part 5/20). Self-organizing maps showing the causes of death of the 16 clusters**

**Figure A4 (part 6/20). Self-organizing maps showing the causes of death of the 16 clusters**

**Figure A4 (part 7/20). Self-organizing maps showing the causes of death of the 16 clusters**

**Figure A4 (part 8/20). Self-organizing maps showing the causes of death of the 16 clusters**

**Figure A4 (part 9/20). Self-organizing maps showing the causes of death of the 16 clusters**

**Figure A4 (part 10/20). Self-organizing maps showing the causes of death of the 16 clusters**

**Figure A4 (part 11/20). Self-organizing maps showing the causes of death of the 16 clusters**

**Figure A4 (part 12/20). Self-organizing maps showing the causes of death of the 16 clusters**

**Figure A4 (part 13/20). Self-organizing maps showing the causes of death of the 16 clusters**

**Figure A4 (part 14/20). Self-organizing maps showing the causes of death of the 16 clusters**

**Figure A4 (part 15/20). Self-organizing maps showing the causes of death of the 16 clusters**

**Figure A4 (part 16/20). Self-organizing maps showing the causes of death of the 16 clusters**

**Figure A4 (part 17/20). Self-organizing maps showing the causes of death of the 16 clusters**

**Figure A4 (part 18/20). Self-organizing maps showing the causes of death of the 16 clusters**

**Figure A4 (part 19/20). Self-organizing maps showing the causes of death of the 16 clusters**

**Figure A4 (part 20/20). Self-organizing maps showing the causes of death of the 16 clusters**
